# Engineering Correlation-Driven Magnetism by Atomic Substitution in Metal-Free Phenalenyl-Based Two-Dimensional Polymers

**DOI:** 10.3390/molecules31050897

**Published:** 2026-03-08

**Authors:** Shiru Yang, Xin Guo, Jing Wang, Bin Shao, Xu Zuo

**Affiliations:** 1 College of Electronic Information and Optical Engineering, Nankai University, Tianjin 300350, China; shiruyang@mail.nankai.edu.cn (S.Y.); guoxkkkk@163.com (X.G.); 1120210137@mail.nankai.edu.cn (J.W.); 2 Tianjin Key Laboratory of Optoelectronic Sensor and Sensing Network Technology, Nankai University, Tianjin 300350, China; 3 Key Laboratory of Photoelectronic Thin Film Devices and Technology of Tianjin, Tianjin 300350, China; 4 Engineering Research Center of Thin Film Optoelectronics Technology, Ministry of Education, Tianjin 300350, China

**Keywords:** metal-free 2D polymers, phenalenyl, correlation-driven magnetism

## Abstract

Metal-free two-dimensional (2D) polymers built from open-shell π-conjugated units offer a promising platform for realizing correlation-driven magnetism without transition metal elements. Here, we present a systematic first-principles study of phenalenyl-based 2D polymers that elucidates how atomic-level chemical substitution controls magnetic order through the interplay of electronic correlation and sublattice symmetry. Combining density functional theory with an effective tight-binding and Hubbard model analysis, we show that atomic substitution with boron or nitrogen on phenalenyl building blocks acts as a sublattice-resolved tuning knob for both the ratio of on-site Coulomb interaction to inter-site hopping (U/t) and the relative on-site energies of the two sublattices. Sublattice-asymmetric substitution with boron or nitrogen breaks sublattice equivalence and drives the system from an antiferromagnetic Mott-insulating state into spin-polarized semiconducting phases with pronounced spin-dependent gaps. In contrast, uniform substitution on both sublattices preserves symmetry and yields nonmagnetic metallic states characterized by rigid band shifts rather than correlation-driven spin polarization. These results establish a unified microscopic framework in which electronic correlations and sublattice symmetry emerge as cooperative yet independently tunable parameters, providing general design principles for metal-free 2D π-conjugated materials with tailored magnetic and spintronic functionalities.

## 1. Introduction

Metal-free two-dimensional (2D) materials with controllable magnetic order hold promise for sustainable spintronic applications [1,2], yet remain challenging to realize in π-conjugated organic systems. The strong delocalization of π-orbital electrons in graphene-like frameworks typically favors nonmagnetic or antiferromagnetically compensated ground states [3,4,5], even when defects or edge states are introduced [6,7]. Identifying chemically accessible mechanisms to achieve tunable magnetism in metal-free 2D polymers therefore remains a fundamental challenge.

Open-shell molecular building blocks, particularly phenalenyl (PLY) radicals with their persistent singly occupied molecular orbitals, offer a promising route toward correlation-driven magnetism in organic materials. Open-shell molecular building blocks provide a promising route toward correlation-driven magnetism in organic materials [8]. Among various organic radicals, phenalenyl (PLY) is particularly attractive because it simultaneously combines intrinsic open-shell character with a threefold symmetric π-conjugated framework that naturally supports extended lattice formation. The PLY radical stabilizes a singly occupied molecular orbital (SOMO) delocalized over a π-conjugated backbone, giving rise to a robust spin-1/2 degree of freedom without involving transition metal ions [9,10,11,12]. Moreover, the symmetry and connectivity of PLY enable its assembly into bipartite honeycomb networks, allowing each molecular SOMO to map naturally onto an effective lattice site and providing a molecular platform for Hubbard-type correlated physics. Recent experimental advances in on-surface synthesis have enabled atomically precise fabrication of PLY- and triangulene-based lattices, revealing antiferromagnetic Mott insulating states and emergent magnetic behavior [13,14,15,16]. However, these studies have largely focused on specific lattice geometries or substitution patterns, and a systematic understanding of how chemical modification controls magnetic order is lacking. Placed within the broader context of two-dimensional magnetism, phenalenyl-based polymers differ fundamentally from transition metal magnets governed by localized d-orbitals [17,18] or moiré systems relying on twist-induced flat bands [19], as magnetism here originates purely from correlated p-orbital electrons. However, a unified understanding of how chemical modification controls magnetic order in such radical-based polymers remains lacking.

From a theoretical perspective, magnetism in π-conjugated networks arises from the interplay between on-site Coulomb repulsion (U) and inter-site hopping (t), captured by Hubbard-type models [20,21]. In bipartite lattices such as honeycomb structures, sublattice symmetry plays a critical role: at half filling, strong correlations (large U/t) drive antiferromagnetic Mott states via superexchange [22,23], while breaking sublattice equivalence can fundamentally alter the magnetic ground state [24,25]. Atomic substitution with heteroatoms—particularly boron and nitrogen, which act as electron acceptors and donors respectively [26,27]—provides a natural means of tuning both carrier concentration and sublattice-resolved potentials. Despite extensive work on heteroatom doping in graphene and related materials [28,29,30,31], the interplay between sublattice-selective substitution and strong electronic correlations in open-shell organic polymers has not been systematically explored.

In this work, we establish a direct connection between atomic substitution, electronic correlations, and magnetic order in PLY-based 2D polymers through systematic first-principles calculations combined with effective Hubbard model analysis. We demonstrate that sublattice-resolved boron or nitrogen substitution provides independent control over correlation strength (U/t) and sublattice potential asymmetry (Δ). Crucially, we show that sublattice-asymmetric substitution—where heteroatoms are introduced selectively on one sublattice—drives a magnetic phase transition from antiferromagnetic Mott insulating states to spin-polarized semiconducting phases with net magnetization. In contrast, uniform substitution preserving sublattice symmetry yields nonmagnetic metallic behavior governed by rigid band shifts. By analyzing projected density of states, spin density distributions, and effective model parameters, we reveal the microscopic mechanism underlying this controllable magnetism and establish design principles for engineering correlation-driven magnetic order in metal-free 2D π-conjugated materials.

## 2. Results and Discussion

**Figure 1:**
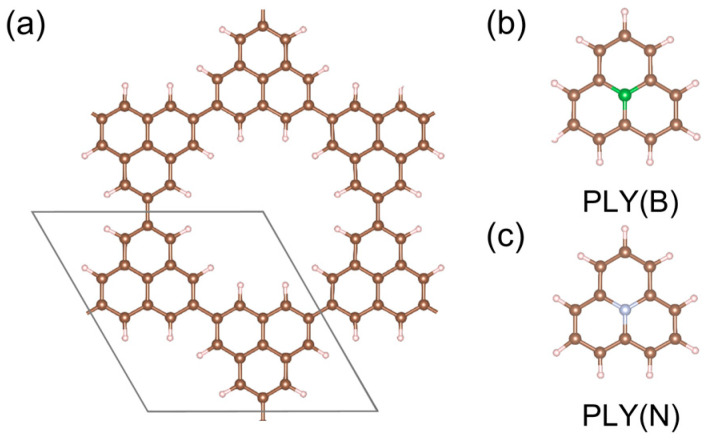
Structures of PLY-based 2D polymers and doped molecular units. (**a**) Optimized atomic structure of the pristine PLY-based 2D polymer. The black parallelogram denotes the primitive unit cell. (**b**,**c**) Molecular structures of the PLY building blocks with central-site substitution by B [PLY(B)] and N [PLY(N)], respectively, which serve as the basic units for constructing sublattice-doped honeycomb frameworks. Carbon, boron, and nitrogen atoms are shown in brown, green, and silver, respectively.

To elucidate the magnetism and electronic tunability in 2D polymers, we constructed an organic radical framework in which PLY units form a covalently linked honeycomb lattice (Figure 1a), that preserves the π-conjugated connectivity. The pristine structure PLY serves as the reference configuration, while targeted atomic substitutions are introduced at the central carbon site to form B/N-functionalized variants (Figure 1b,c), respectively.

**Figure 2:**
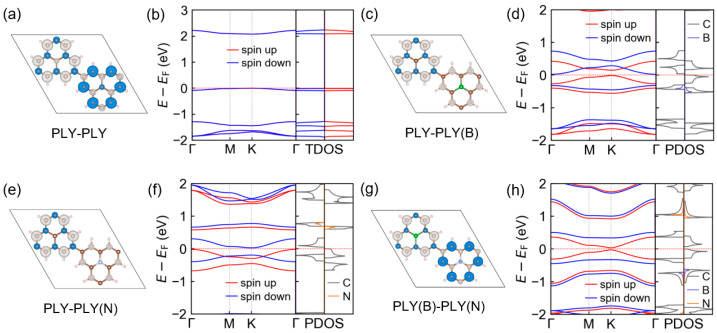
Spin density and electronic structures of PLY-based polymers with B/N substitution. (**a**,**c**,**e**,**g**) Spin-density distributions of the primitive unit cells for different molecular fillings on the A and B sublattices: pristine [PLY–PLY], B-substituted [PLY–PLY(B)], N-substituted [PLY–PLY(N)], and charge-compensated [PLY(B)–PLY(N)], respectively. Spin-up and spin-down components are shown in white and blue, respectively. The pristine [PLY–PLY] and charge-compensated [PLY(B)–PLY(N)] systems exhibit fully compensated spin configurations with zero net magnetic moment, whereas [PLY–PLY(B)] and [PLY–PLY(N)] display uncompensated spin-density distributions. (**b**,**d**,**f**,**h**) Corresponding spin-resolved band structures and total/projected density of states (TDOS/PDOS) for [PLY–PLY], [PLY–PLY(B)], [PLY–PLY(N)], and [PLY(B)–PLY(N)], respectively. The red dashed line denotes the Fermi level.

For the [PLY–PLY] system (Figure 2a,b), the spin density is fully compensated between the two molecular sublattices, and the spin-up and spin-down electronic structures remain identical throughout the Brillouin zone. The low-energy π-derived bands are weakly dispersive and separated by a finite gap, consistent with an antiferromagnetic Mott-insulating ground state of a half-filled correlated π network, with the corresponding quantitative values summarized in Supplementary Table S1.

Substitution with a hole-rich B center or an electron-rich N center leads to a pronounced localization of an uncompensated spin on the substituted PLY unit, while the remaining sublattice retains a largely compensated spin character (Figure 2c,e). This behavior directly reflects a dopant-induced modification of the local electron count and a redistribution of spin density across the conjugated backbone. The spin-resolved band structures (Figure 2d,f) exhibit pronounced dopant-dependent shifts in the low-energy bands, consistent with hole-type (B) and electron-type (N) doping at the molecular level. Importantly, B or N substitution induces a global spin-dependent reorganization of the π-derived electronic states, which renders the two spin channels inequivalent and produces distinct gap magnitudes, thereby establishing half-semiconducting states in both [PLY–PLY(B)] and [PLY–PLY(N)]. The resulting electronic structure is accompanied by a net magnetic moment of 1 μB per primitive cell.

A qualitatively different situation emerges for the charge-compensated [PLY(B)–PLY(N)] configuration. With opposite spin polarizations residing on the B- and N-substituted units, the total magnetic moment of the unit cell is fully compensated (Figure 2g). Despite the absence of a net moment, the low-energy π bands exhibit half-semiconducting behavior, characterized by a nearly gapless channel for one spin species while the other maintains a finite gap (Figure 2h). This coexistence of global spin compensation and pronounced spin selectivity highlights the unique role of sublattice-resolved B/N co-doping in reshaping the π-electronic structure and establishes a favorable electronic landscape for spin-selective transport under external modulation. Further insight is provided by the total and projected density of states, which shows that the electronic states governing these spin-dependent features are dominated by the π-conjugated carbon framework, whereas B- and N-derived contributions are largely confined to deeper energy regions. This confirms that B/N substitution primarily modulates the spin and charge distribution of the extended π network, rather than introducing isolated impurity states. Although the low-energy bands resemble spin-resolved massive Dirac bands, the gap in the present system mainly arises from sublattice symmetry breaking and spin polarization rather than strong spin–orbit coupling or complex hopping phases typically associated with topological band formation [32,33,34]. Under these conditions, pronounced topological effects are not anticipated to dominate the low-energy physics. Nevertheless, a detailed analysis of Berry curvature and possible topology-dependent responses would require a dedicated investigation beyond the scope of the present work.

Although sublattice symmetry is modified by atomic substitution, the electronic structures at the K and K′ points remain symmetry-related in the present system, as shown in Figure S2 of the Supplementary Materials. In the absence of spin–orbit coupling, time-reversal symmetry connects the electronic states at K and K′, such that no pronounced valley-dependent spin splitting is expected. As a result, the net magnetic moment is determined by the global spin imbalance rather than valley-dependent band splitting. This situation differs fundamentally from transition metal dichalcogenides, where strong spin–orbit coupling combined with inversion symmetry breaking leads to inequivalent spin-split valleys. To explicitly verify this point, a comparison of the band structures along the K and K′ directions has been provided in the Supplementary Materials, confirming their equivalence within numerical accuracy.

**Figure 3:**
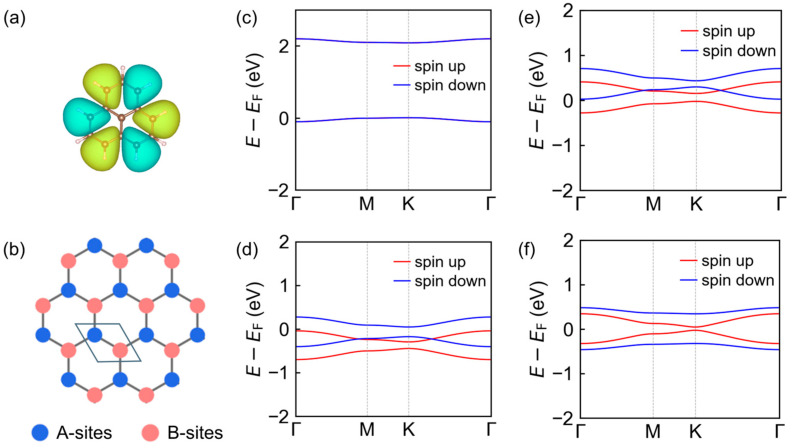
Single-orbital tight-binding description of PLY-based honeycomb frameworks. (**a**) Real-space wavefunction of the singly occupied molecular orbital (SOMO) of an isolated PLY molecule, illustrating the spatial distribution of the unpaired π electron. (**b**) Schematic representation of the PLY-based framework mapped onto an effective honeycomb lattice, where each PLY unit is treated as a lattice site; blue and red circles denote the two inequivalent sublattices (A and B), respectively (**c**–**f**). Spin-resolved band structures obtained from single-orbital tight-binding model fits for different PLY-based configurations: (**c**) pristine PLY–PLY, (**d**) PLY–PLY(N), (**e**) PLY–PLY(B), and (**f**) PLY(B)–PLY(N).

To interpret the spin-resolved electronic structures obtained from first-principles calculations, we construct a minimal single-orbital tight-binding description based on the PLY building block. As shown in Figure 3a, the singly occupied molecular orbital (SOMO) of an isolated PLY molecule is spatially extended over the π-conjugated backbone, justifying its treatment as an effective low-energy degree of freedom. On this basis, each PLY unit is mapped onto a lattice site of an effective honeycomb network, with the two molecular sublattices labeled as A and B (Figure 3b). As shown in Figure 3a, the singly occupied molecular orbital (SOMO) of an isolated PLY molecule is spatially extended over the π-conjugated backbone. Electronic structure analysis indicates that the bands near the Fermi level predominantly originate from frontier π states of the PLY units, allowing this orbital to serve as the effective molecular orbital associated with each PLY site. Each PLY unit is therefore mapped onto a lattice site of an effective honeycomb network, with the two molecular sublattices labeled as A and B (Figure 3b). The Hamiltonian is written as

(1) $$\hat{H}={\hat{H}}_{0}+{\hat{H}}_{U},$$

where the single-particle term reads

(2) $${\hat{H}}_{0}=\sum_{i\in A,\sigma }\varepsilon_{A}{\hat{n}}_{i\sigma }+\sum_{j\in B,\sigma }\varepsilon_{B}{\hat{n}}_{j\sigma }-t\sum_{\left\langle i\in A,j\in B\right\rangle ,\sigma }({\hat{c}}_{i\sigma }^{†}{\hat{c}}_{j\sigma }+h.c.),$$

where ${\hat{c}}_{l}^{†}({\hat{c}}_{l})$ creates (annihilates) an electron with spin σ on site *l*, ${\hat{n}}_{l\sigma }={\hat{c}}_{l\sigma }^{†}{\hat{c}}_{l\sigma },$ t denotes the nearest-neighbor hopping between A and B sublattices, and $\varepsilon_{A}$ and $\varepsilon_{B}$ are effective on-site energies that account for sublattice inequivalence, for instance induced by B/N substitution.

Electron-electron interactions within each effective orbital are described by an on-site Hubbard term

(3) $${\hat{H}}_{U}=U\sum_{l}{\hat{n}}_{l↑}{\hat{n}}_{l↓}.$$

Within the mean-field approximation, the effective spin-dependent Hamiltonian in momentum space reads

(4) $${\hat{H}}_{\sigma }\left(k\right)=\left(\begin{array}{cc} \varepsilon_{A\sigma }^{eff} & -tf\left(k\right)\\ -tf^{*}\left(k\right) & \varepsilon_{B\sigma }^{eff} \end{array}\right),$$

with the structure factor $f\left(k\right)=\sum_{j=1}^{3}e^{ik\cdot \delta_{j}}$, where $\left\{\delta_{j}\right\}$ are the three nearest-neighbour vectors from an A site to its three B neighbours, and spin-dependent effective on-site energies $\varepsilon_{A\sigma }^{eff}=\varepsilon_{A}+Un_{A\bar{\sigma }}$, $\varepsilon_{B\sigma }^{eff}=\varepsilon_{B}+Un_{B\bar{\sigma }}$, where $n_{A\bar{\sigma }}$ and $n_{B\bar{\sigma }}$ denote the spin-resolved occupations on the A and B sublattices, respectively, and $\bar{\sigma }$ indicates the opposite spin.

The tight-binding models reproduce the evolution of the spin-resolved electronic structures upon B/N substitution in PLY-based polymers, with the corresponding model parameters for each configuration summarized in Table 1. For the [PLY-PLY] framework, the fitted tight-binding bands remain spin degenerate and reproduce the characteristic low-energy dispersion (Figure 3c). The extracted interaction ratio U/t=12.6 places this half-filled system deep in the strongly correlated regime. In the canonical half-filled honeycomb Hubbard model, quantum Monte Carlo studies have established a critical coupling $U_{c}/t\approx 3.8$ separating semimetallic and antiferromagnetic Mott-insulating states [35,36], and larger U/t values favor electron localization and magnetic ordering. The sublattice potential difference is defined as $\;\Delta \varepsilon =\;\varepsilon_{A}-\varepsilon_{B}$, where A and B denote the two molecular sublattices of the effective honeycomb lattice. Throughout this work, the structural notation X-Y follows the sublattice assignment such that the first and second molecular units correspond to the A and B sublattices, respectively. Accordingly, in the PLY(B)-PLY(N) structure the A sublattice is occupied by B-substituted PLY units, while the B sublattice is occupied by N-substituted PLY units. The same convention applies to other configurations, such as PLY-PLY(B) and PLY-PLY(N). The parameter Δε therefore represents the relative alignment of the effective onsite energies associated with the two molecular sublattices rather than an absolute energy change. The absence of sublattice asymmetry (Δε=0) in this case preserves the equivalence of the two sublattices, leading to a globally compensated spin distribution consistent with an antiferromagnetic Mott-correlated insulator.

Introduction of substitution breaks this symmetry and modifies both the local potential and effective filling of the π network. The reduction in *U* upon substitution reflects that sublattice asymmetry (Δ*ε*) partially accounts for the low-energy band reconstruction, reducing the interaction strength required within the tight-binding model. In the [PLY–PLY(N)] configuration, the fitted ratio U/t=5.55 and nonzero on-site offset Δε=−0.22 eV reflect a reduction in effective correlation strength relative to the pristine case and a significant sublattice inequivalence. In this intermediate-to-strong correlated regime, the competition between kinetic processes and Coulomb repulsion in the presence of sublattice potential difference drives a spin-resolved reconstruction of the low-energy spectrum, yielding an uncompensated net moment ($M=1\mu_{B}$). A closely analogous picture emerges for the [PLY–PLY(B)] system, with U/t=5.45 and Δε=−0.18eV. The slight shift in Δε associated with the hole-rich B center produces a distinct band reorganization (Figure 3d,e), yet both substituted cases remain strongly correlated on the honeycomb lattice while manifesting sublattice-driven spin polarization.

The charge-compensated [PLY(B)–PLY(N)] configuration further illustrates the interplay between sublattice asymmetry and strong electronic correlations. The fitted parameters U/t=5.8 and a larger on-site offset Δε=−0.50eV correspond to a situation in which the low-energy electronic bands exhibit clear spin splitting while remaining globally compensated (Figure 3f). This arises from the opposite chemical potentials associated with B- and N-substituted sublattices, which drive sublattice-resolved spin splitting in the low-energy π bands while preserving global spin compensation.

Collectively, the fitted tight-binding parameters and resulting band reconstructions underscore that all four configurations lie in a regime dominated by electron correlation, well above the critical coupling identified in canonical honeycomb Hubbard systems. Sublattice-resolved modifications of the effective on-site energies and fillings act as relevant perturbations in this correlated regime, selecting distinct spin-resolved electronic responses depending on the substitution pattern. This framework provides a coherent context for interpreting the first-principles electronic structures and highlights the utility of interaction-to-hopping ratios and sublattice asymmetry as organizing principles for spin order in PLY-based π networks.

**Figure 4:**
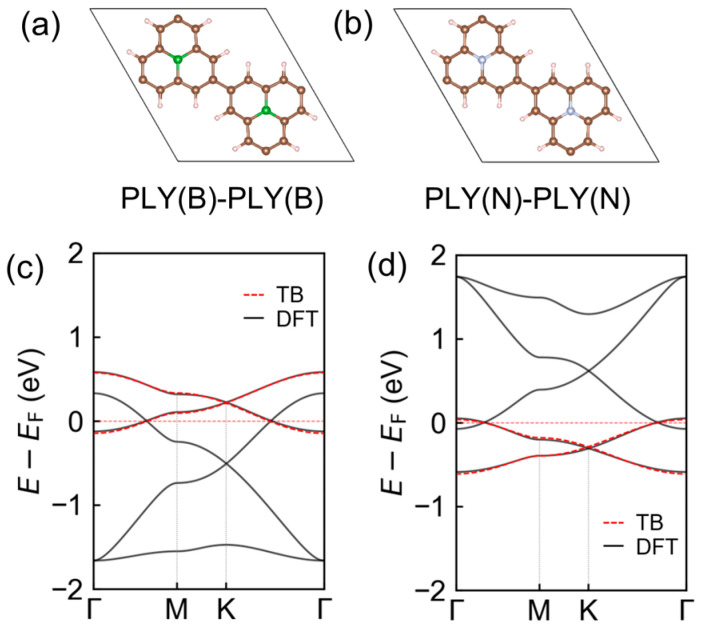
Electronic structures of uniformly substituted [PLY(B)-PLY(B)] and [PLY(N)-PLY(N)] frameworks. (**a**,**b**) Optimized unit-cell geometries of uniformly substituted [PLY(B)-PLY(B)] and [PLY(N)-PLY(N)] frameworks. (**c**,**d**) Corresponding electronic band structures.

In contrast to the sublattice-asymmetric substitution patterns discussed above, uniform substitution at both A and B sites in the [PLY(B)–PLY(B)] and [PLY(N)–PLY(N)] frameworks (Figure 4a,b) preserves sublattice equivalence and leads to complete charge compensation, resulting in a nonmagnetic state. In these uniformly substituted systems, the fitted nearest-neighbor hopping remains essentially unchanged (t = 0.11 eV), indicating that the overall π-network connectivity and bandwidth are largely unaffected by the modest chemical modification. The corresponding band structures obtained from both tight-binding fits and first-principles calculations (Figure 4c,d) display metallic characteristics with a Dirac-like linear band crossing retained in the low-energy spectrum. Importantly, this crossing is shifted rigidly in energy relative to the Fermi level, reflecting a change in band filling rather than a reconstruction of the underlying electronic structure. Uniform boron substitution introduces hole-like carriers and shifts the Dirac crossing to lower energies, whereas nitrogen substitution donates electrons and moves it upward. Throughout this process, spin degeneracy is fully preserved, consistent with the absence of sublattice imbalance and net magnetic moment.

The sublattice-selective magnetic control demonstrated here opens promising pathways for metal-free spintronics and quantum materials. Experimental realization through on-surface synthesis of B/N-functionalized PLY precursors on catalytic substrate would enable direct STM/STS verification of predicted spin-polarized states. The half-semiconducting configurations exhibit intrinsic spin-filtering behavior ideal for all-organic spin valves and gate-tunable magnetic memory, circumventing conductivity mismatch challenges in conventional metal–organic heterostructures.

The strong correlations (U/t > 5) position these systems at the threshold of exotic quantum phases. Extension to larger polycyclic radicals (triangulene, starphene) could realize quantum spin liquids or topologically nontrivial magnon bands. Incorporating heavier heteroatoms may induce spin–orbit coupling, potentially yielding quantum anomalous Hall states or higher-order topological insulators. Achieving room-temperature magnetism through 3D covalent frameworks or magnetic proximity effects with established 2D ferromagnets remains critical for practical spintronic applications. The physically transparent U/t -Δ framework established here provides experimentally accessible design principles for correlation-driven magnetism in π-conjugated materials.

All calculations in this work are performed for free-standing frameworks to reveal the intrinsic correlation-driven magnetic interactions. In realistic experimental conditions, however, PLY-based two-dimensional polymers are typically synthesized on substrates, where Fermi-level alignment may induce partial charge transfer and electronic redistribution.

Such substrate-induced doping is known to modify magnetic ordering in low-dimensional systems by changing orbital occupation and exchange interactions, as demonstrated in gated two-dimensional magnets [18]. Within this picture, the magnetic states obtained here represent the charge-neutral reference limit, while moderate electron or hole transfer from the substrate may tune the relative stability between competing magnetic configurations. In particular, small deviations from charge neutrality could lift the spin compensation in the PLY(N)–PLY(B) framework and stabilize a finite magnetic moment. Therefore, substrate effects are expected to provide an experimentally accessible tuning mechanism rather than fundamentally altering the correlation-driven magnetism identified in this work.

Although the alternating B- and N-substituted PLY lattices proposed here have not yet been experimentally realized, their design is consistent with recent progress in bottom-up nanographene synthesis [37]. Heteroatom substitution in PLY-based π-conjugated systems, including nitrogen-substituted PLY analogues [38] and boron-doped polycyclic aromatic hydrocarbons [39], demonstrates the accessibility of chemically stable substituted building blocks. Among possible fabrication strategies, on-surface synthesis provides the most direct near-term route, where heteroatom-programmed molecular precursors may undergo surface-assisted coupling and cyclodehydrogenation to form atomically precise two-dimensional networks. The present structures should therefore be regarded as experimentally motivated design targets that offer guiding principles for future realization of metal-free magnetic two-dimensional polymers.

## 3. Computational Methods

DFT calculations were performed using the projector augmented wave (PAW) [40] method as implemented in the Vienna Ab Initio Simulation Package (VASP, version 5.4.4; University of Vienna, Vienna, Austria) [41]. Electron exchange and correlation were treated using The generalized gradient approximation (GGA) [42] as parameterized by Perdew–Burke–Ernzerhof (PBE) functional have been widely employed to investigate the electronic and magnetic properties of π-conjugated organic frameworks, particularly in systems with appreciable electronic delocalization, such as the B/N-substituted lattices considered here. In contrast, hybrid-functional calculations employing the PBE0 functional (incorporating 25% exact Hartree–Fock exchange) were performed for the pristine PLY–PLY lattice, for which hybrid functionals provide an improved description of localized open-shell states and correlation-enhanced gap formation, as demonstrated in previous benchmark studies of related π-radical 2D polymers [43,44,45]. For the pristine PLY–PLY lattice, we performed a direct comparison between PBE and PBE0 (Supplementary Material Figure S1), the results show that both functionals predict the same magnetic ground state and qualitatively consistent low-energy band features. The primary difference lies in the magnitude of the band gap, which is enhanced under PBE0, while the overall magnetic ordering and gap character remain unchanged. This preservation of magnetic ordering and electronic classification across functionals indicates that the phase identification and spin-resolved electronic features discussed in the main text are robust with respect to the choice of exchange–correlation functional. To accurately capture the electronic correlation effects characteristic of open-shell PLY radical systems, hybrid functional calculations employing the PBE0 functional (incorporating 25% exact Hartree–Fock exchange) were additionally performed for selected configurations. The PBE0 functional has been demonstrated to provide superior descriptions of localized magnetic states and correlation-driven phenomena in π-conjugated systems compared to standard GGA functionals [44,45].

The plane-wave kinetic energy cutoff was set to 500 eV, which ensures total energy convergence to within 1 meV per atom as verified by systematic convergence tests. All atomic structures were fully relaxed using the conjugate gradient algorithm until energy changes between successive ionic steps were below 10^−5^ eV and residual forces on each atom were less than 0.01 eV/Å. Brillouin zone integrations were sampled using a Γ-centered Monkhorst-Pack k-point mesh with a density of 5 × 5 × 1 for structural optimization and 6 × 6 × 1 for high-precision electronic structure calculations and density of states analysis. A vacuum layer of at least 25 Å was employed in the direction perpendicular to the 2D slab to eliminate spurious interactions between periodic images, confirmed by negligible interlayer binding energies.

The pristine PLY-based 2D polymer was constructed by covalently linking PLY (C_13_H_9_) radical units through carbon-carbon bonds in a honeycomb lattice arrangement. The minimal unit cell contains two PLY units corresponding to the A and B sublattices of the bipartite honeycomb structure. Initial atomic coordinates were generated based on standard bond lengths for polycyclic aromatic hydrocarbons (C-C: 1.42 Å, C-H: 1.09 Å), and the lattice parameters were fully optimized without symmetry constraints, yielding optimized lattice constants of a = b ≈ 12.306 Å with hexagonal symmetry (γ = 120°). For each substitution pattern, all atomic positions were re-optimized to account for local structural relaxation induced by the size and electronegativity differences in the heteroatoms.

Tight-binding parameters were extracted by fitting the Hubbard mean-field band structure to DFT results using the L-BFGS-B algorithm implemented in Python 3.14.3 (Python Software Foundation, Wilmington, DE, USA). The objective function minimized a weighted sum of point-wise mean-square deviation, bandwidth mismatch, shape dissimilarity, energy alignment error, and spin-splitting difference between tight-binding and DFT bands. The relative weights of these terms were adjusted for each system to ensure balanced reproduction of the low-energy DFT band features. The practical uncertainties associated with the fitted parameters are estimated from their sensitivity to reasonable variations in fitting initialization and loss-function weighting. These uncertainties are small compared with the substitution-induced parameter changes summarized in Table 1, supporting the robustness of the extracted interaction and hopping trends.

## 4. Conclusions

In conclusion, our combined DFT and tight-binding analysis establishes sublattice-resolved atomic substitution as a central control parameter for correlation-driven spin order in PLY-based π-conjugated polymers. The pristine [PLY–PLY] framework resides in a strongly correlated Mott-singlet regime at large U/t, whereas sublattice-selective substitution with boron or nitrogen breaks sublattice equivalence and drives the system intro spin-polarized semiconducting states. In contrast, uniform B–B and N–N substitution preserves sublattice symmetry and yields nonmagnetic metallic state, underscoring that carrier doping alone is insufficient to generate magnetism in these systems. Charge-compensated co-doping further demonstrates that local spin polarization and global magnetization can be independently controlled. Taken together, these results establish a unified microscopic picture in which the interplay between electronic correlations and sublattice potential asymmetry governs magnetic responses in metal-free π systems, offering general design principles for organic spin-functional materials.

## Figures and Tables

**Table 1:** Magnetic properties of PLY-based 2D polymers. Calculated on-site Coulomb interaction U, hopping parameter t, their ratio U/t, on-site potential difference Δε, and net magnetic moment M for the investigated PLY-based lattice configurations.

	U (eV)	|t| (eV)	U/t	Δ*ε* = ε_A_ − ε_B_	M (μB)
PLY-PLY	2.15	0.17	12.6	0.00	0.0
PLY-PLY(B)	0.60	0.11	5.45	−0.18	1.0
PLY-PLY(N)	0.61	0.11	5.55	−0.22	1.0
PLY(B)-PLY(N)	0.64	0.11	5.80	−0.50	0.0

## Data Availability

All data supporting this study are included in the article.

## Supplementary Materials

The following supporting information can be downloaded at: https://www.mdpi.com/article/10.3390/molecules31050897/s1, Figure S1: Spin-polarized band structures and TDOS of the PLY–PLY dimer (PBE0 and PBE); Table S1: Spin-resolved band gaps, gap character, k-point locations, and density of states at the Fermi level for the PLY-based lattice configurations shown in Figure 2; Figure S2: Spin-resolved band structures of PLY-based configurations along M–K–Γ–K′–M.
