# Supplementary material for: Engineering Correlation-Driven Magnetism by Atomic Substitution in Metal-Free Phenalenyl-Based Two-Dimensional Polymers
Source: Molecules. 2026 Mar 8;31(5):897. doi: 10.3390/molecules31050897 (PMC12985530; doi:10.3390/molecules31050897)
Supplement: Supplementary file 1 [file molecules-31-00897-s001.zip › molecules-4164483-supplementary.pdf]

# Engineering Correlation-Driven Magnetism by Atomic Substitution in Metal-Free Phenalenyl-Based Two-Dimensional Polymers

**Shiru Yang [1], Xin Guo [1], Jing Wang [1], Bin Shao [1,2,*] and Xu Zuo [1,3,4,*]**

1 College of Electronic Information and Optical Engineering, Nankai University, Tianjin 300350, China; shiruyang@mail.nankai.edu.cn (S.Y.); guoxkkkk@163.com (X.G.); 1120210137@mail.nankai.edu.cn (J.W.)
2 Tianjin Key Laboratory of Optoelectronic Sensor and Sensing Network Technology, Nankai University, Tianjin 300350, China
3 Key Laboratory of Photoelectronic Thin Film Devices and Technology of Tianjin, Tianjin 300350, China
4 Engineering Research Center of Thin Film Optoelectronics Technology, Ministry of Education,
Tianjin 300350, China
* Correspondence: bshao@nankai.edu.cn (B.S.); xzuo@nankai.edu.cn (X.Z.)

To further clarify the electronic structure of the [PLY–PLY] system, we calculated the spin-polarized band structures and total density of states using both PBE and PBE0 functionals. As shown in Figure S1, both methods yield fully spin-degenerate solutions. The spin-up and spin-down bands overlap throughout the entire Brillouin zone, and the corresponding TDOS exhibits symmetric contributions from the two spin channels, confirming the absence of net spin polarization in the ground state.

Importantly, both functionals predict a finite energy gap at the Fermi level, with the density of states vanishing at $E_F$. While the overall electronic character remains qualitatively consistent between the two functionals, a quantitative difference is evident in the gap magnitude. The PBE0 functional yields a noticeably larger gap compared to PBE. Taken together, both PBE and PBE0 consistently describe a spin-degenerate insulating ground state, with the hybrid functional predicting a stronger effective correlation effect reflected in the enlarged gap.

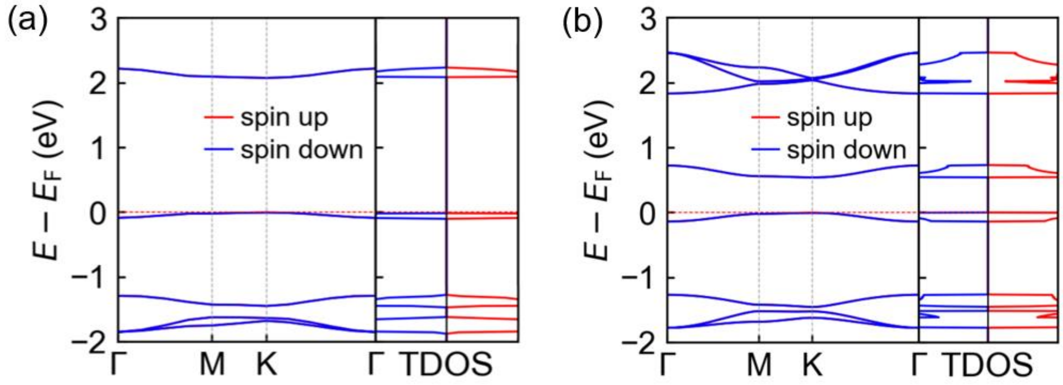


Figure S1. Spin-polarized electronic band structures and total density of states (TDOS) of the PLY–

PLY dimer calculated using (a) PBE0 and (b) PBE functionals. The red and blue curves represent spin-up and spin-down channels, respectively.

The spin-resolved band gaps were independently extracted from the calculated band structures along the high-symmetry k-path. For each spin channel, the valence-band maximum and conduction-band minimum occur at the same k-point, confirming direct-gap character in all configurations.

For the pristine PLY–PLY lattice, both spin channels exhibit identical direct band gaps located at the K point, consistent with an antiferromagnetic Mott-insulating ground state of a half-filled correlated π network.

For the substituted [PLY–PLY(B)] and [PLY–PLY(N)] configurations, each spin channel retains a direct band gap; however, the gap magnitudes differ between spin-up and spin-down states, and their extrema occur at distinct high-symmetry k-points (K and M, respectively). This spin-dependent gap asymmetry is characteristic of half-semiconducting behavior in both systems.

In contrast, for the [PLY(B)–PLY(N)] configuration, the band extrema of both spin channels occur at the same high-symmetry point (K), but the gap magnitudes remain unequal, with the spin-up gap reduced to as small as 0.07 eV. These features indicate that sublattice-resolved B/N co-doping significantly reshapes the low-energy π-electronic structure, leading to a spin-asymmetric half-semiconducting state that may be relevant for spin-selective transport under external modulation. The vanishing density of states at the Fermi level in both spin channels further confirms that all four configurations remain electronically gapped.

Table S1. Spin-resolved band gaps (Eg↑, Eg↓), gap character, corresponding k-point locations, and density of states at the Fermi level for the PLY-based lattice configurations shown in Figure 2.

| **Configuration** | **Eg↑ (eV)** | **Eg↓ (eV)** | **Gap type** | **k-point↑** | **k-point↓** | **DOS↑($E_F$)** | **DOS↓($E_F$)** |
|---|---|---|---|---|---|---|---|
| **PLY-PLY** | 2.06 | 2.06 | direct | K | K | 0.00 | 0.00 |
| **PLY-PLY(B)** | 0.16 | 0.34 | direct | K | Γ | 0.00 | 0.00 |
| **PLY-PLY(N)** | 0.60 | 0.21 | direct | Γ | K | 0.00 | 0.00 |
| **PLY(B)-PLY(N)** | 0.07 | 0.66 | direct | K | K | 0.00 | 0.00 |

As shown in Figure S2, the band dispersions at the K and K′ points are symmetry equivalent for all considered configurations. Although sublattice asymmetry is introduced via chemical substitution, the calculations are performed within a collinear spin framework without spin–orbit coupling. Under these conditions, time-reversal

symmetry relates the electronic structures at K and K′, resulting in identical energy dispersions without valley-dependent splitting. Consequently, no independent valley polarization emerges, and the net magnetic moment arises from the overall spin imbalance rather than valley-resolved band features.

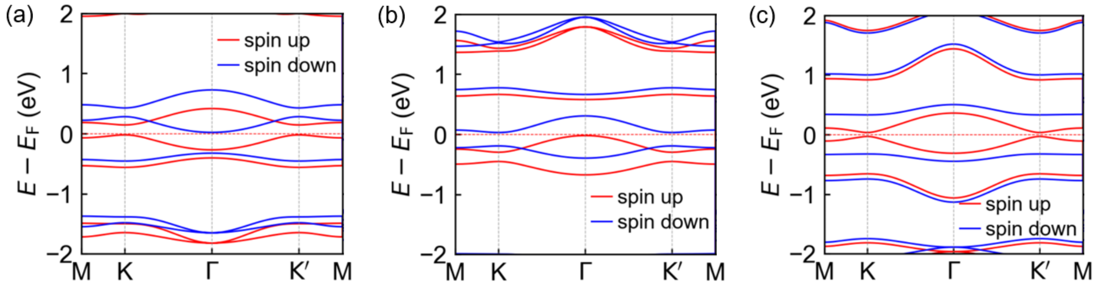


Figure S2. Spin-resolved band structures of (a) [PLY–PLY(B)], (b) [PLY–PLY(N)], and (c) [PLY(B)–PLY(N)] configurations along the high-symmetry path M–K–Γ–K′–M. Red and blue curves represent spin-up and spin-down channels, respectively.
